# WSX1 Expression in Tumors Induces Immune Tolerance via Suppression of Effector Immune Cells

**DOI:** 10.1371/journal.pone.0019072

**Published:** 2011-04-29

**Authors:** Denada Dibra, Jeffry Cutrera, Xueqing Xia, Shulin Li

**Affiliations:** Pediatrics Research Department, MD Anderson Cancer Center, Houston, Texas, United States of America; Centre de Recherche Public de la Santé (CRP-Santé), Luxembourg

## Abstract

Crosstalk between tumor cells and the cognate microenvironment plays a crucial role in tumor initiation and progression. However, only a few genes are known to affect such a crosstalk. This study reveals that WSX1 plays such a role when highly expressed in tumor cells. The expression of WSX1 in Lewis Lung Carcinoma (LLC) and the melanoma cell line AGS induces the death of T cells and inhibits the production of the effector cytokine IFNγ from NK and T cells, resulting in the promotion of tumor growth. These pro-tumorigenic properties of WSX1 are independent of IL27. This key observation reveals a new pathway of tumor-host interaction, which will ultimately lead to better strategies in immune therapy to reverse tumor tolerance.

## Introduction

Tumor initiation and progression are dependent on both aberrant gene expression in tumor cells and the communication between tumor cells and their microenvironment. Accumulating evidence in the past decade has shown that the complex relationship between tumors and immune cells is not a one way street [1], [2]. Studies have found that while some immune cells can destroy transformed cells, other types of immune cells in the tumor microenvironment can promote escape of tumor cells from the immune system and the transition from a pro-inflammatory immune response into a tolerant state [3]. Multiple tumors have adopted such pathways to downregulate the immune response, thus facilitating tumor growth and immune evasion of tumor cells.

Many tumor suppressor genes and oncogenes have been characterized to suppress or promote tumor growth, but fewer genes in tumors are characterized to interact with immune cells, suppress immune cell activities, or trigger tumor tolerance. These few genes, known as suppressing effector immune cells include: upregulation of program death ligand 1 (PD-L1), indoleamine 2,3-dioxygenase (IDO), galectin 3, Fas ligand and so forth [4], [5], [6], [7], [8], [9]. Revealing other crucial immune suppressor genes that affect the communication network between tumor and immune cells will ultimately result in effective anti-cancer therapies.

The current literature exclusively indicates that the function of the IL27 receptor, WSX1, is exclusively associated with IL27 and IL27's signaling in immune cells. IL27-independent function of WSX1 and its association with cancer biology is unknown. One reason is that WSX1 is primarily known to be expressed in immune cells such as monocytes, dendritic cells, T and B lymphocytes, NK, and mast cells [10]. Another reason, perhaps, is that WSX1 needs to pair with gp130 to constitute a functional signal-transducing receptor for IL27 signaling, and the absence of either subunit attenuates IL27-mediated signaling [11]. Recently, we have found that WSX1 is also expressed in multiple types of tumor cell lines and that WSX1 has an important role not only in immune cells but also in cancers of epithelial origin via an IL27-signaling-independent pathway [12]. This discovery is further supported by a recent report which revealed that WSX1 is expressed in another type of epithelial tumor such as melanoma cells [13].

Our previous findings show that many of the human cell lines have a high level of WSX1 expression when compared to a normal control cell line, NCM460, suggesting that WSX1 might promote tumor growth [12]. Using genetically modified tumor cells, we present evidence that the overexpression of WSX1 in two independent tumor models, such as aggressive Lewis Lung Carcinoma (LLC) and melanoma cell line AGS, promotes tumor growth independent of IL27 signaling. The underlying mechanism by which WSX1 promotes tumor growth is dependent on the presence of an immune surveillance system and the pro-tumorigenic properties of WSX1 are independent of IL27. This observation is associated with the induction of immune tolerance via apoptosis of T cells and the reduction of IFNγ in the tumor microenvironment. This discovery reveals a novel mechanism on how tumor and immune cells communicate to regulate the tumor microenvironment and ultimately tumor progression.

## Results

### The paradoxical functions of WSX1 between *in-vivo* and *in-vitro* assays

**Figure 1:**
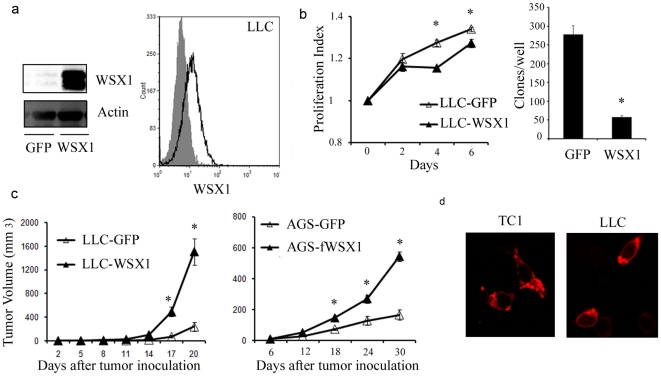
The paradoxical functions of WSX1 between *in-vivo* and *in-vitro* assays. (**a**) Detection of WSX1 expression via western blot (WB) analysis (left) or flow cytometry (right) from LLC tumor cells transduced with retrovirus containing either control GFP or WSX1 gene. Cell extracts of the established cell lines were analyzed using western blot techniques and probed with mouse WSX1 and actin antibodies. (**b**) Detection of the role of WSX1 in tumor cell proliferation. GFP or WSX1 positive LLC cells were harvested on the indicated days and were analyzed for ATP release (left, N = 4, error bars are smaller than symbols). Comparative analysis of soft agar growth assay between LLC-GFP and LLC-WSX1 cells (right, data is representative of two independent experiments, each experiment performed in triplicate). (**c**) Comparison of tumor growth between LLC-GFP and LLC-WSX1 (left, N = 4, representative of four independent experiments) or AGS-GFP and AGS-fWSX1 (right, N = 5). (**d**) Detection of WSX1 localization in the cell. Images of TC1-WSX1 (left) or LLC-WSX1 (right) cells expressing WSX1 gene were captured using a confocal microscope. Points, mean; bars, SE. *, P<0.05 comparing GFP- to WSX1-positive tumors.

We have previously shown that WSX1 is highly expressed not only in immune cells as reported by others, but also in epithelial tumor cells [10], [12]. The expression of WSX1 in tumor cells reduced tumorigenicity and cell proliferation *in-vitro* in two different cell lines, AT84 and TC1. We aimed to further extend this discovery and determine whether WSX1 has the same function in Lewis Lung Carcinoma (LLC). First, we confirmed WSX1 expression via western blot and flow cytometry in LLC cells engineered to express either WSX1 or control GFP (Figure 1a, left and right panels, respectively). The ATP light and clonogenic assays of the genetically engineered LLC cells confirmed our findings in the previous two types of tumor cells: LLC-WSX1 cells grew significantly slower than the cognate control LLC-GFP cells and resulted in a 5-fold reduction in clonogenic ability when compared to LLC-GFP (Figure 1b, left and right panels, respectively).

To test whether the same result would occur *in-vivo*, we compared the tumor growth difference between GFP- or WSX1-positive LLC tumor cells in C57Bl/6 mice. Contrary to the *in-vitro* results and the results of the other two tumor models [12], WSX1 promoted tumor growth in LLC (Figure 1c, left). Similarly, WSX1 enhanced tumor growth in another independent tumor model, melanoma cell line AGS (Figure 1c, right).

To determine how WSX1 promotes LLC tumor *in-vivo*, the WSX1 expression was examined for the abnormal localization in the cell, which may have accounted for tumor promotion. The confocal microscope results ruled out this possibility. Similar to TC1-WSX1 cells; WSX1 was expressed on cell membrane in LLC-WSX1 cells (Figure 1d).

### WSX1 promotes tumor growth independently of IL27

**Figure 2:**
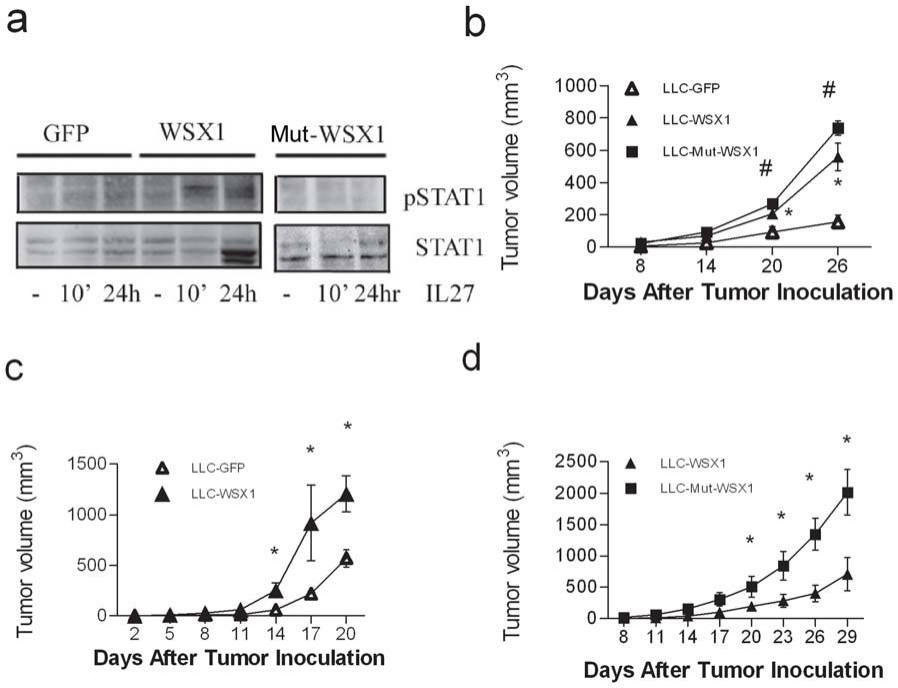
IL27-independent and WSX1-dependent tumor growth promotion. (**a**) Comparison of IL27 signaling function between WSX1- and DN-WSX1 cells. Equal amounts of cell extracts of LLC cells expressing GFP, WSX1, or DN-WSX1 were treated with IL27 for 10 minutes (10′), overnight (24 hr), or left untreated (-), and then analyzed using WB techniques and probed with pSTAT1 and STAT1 antibodies. (**b**) Comparison of tumor growth between LLC-GFP, LLC-WSX1, and LLC-MUT in wildtype C57Bl/6 mice (N = 5). Representative of two independent experiments. (**c**) Comparison of tumor growth between LLC-GFP and LLC-WSX1 in TCCR^−/−^ mice (N = 4). (**d**) Comparison of tumor growth between LLC-WSX1 and LLC-MUT in TCCR^−/−^ mice (N = 4). Points, mean; bars, SE. *, P<0.05 between LLC-WSX1 and LLC-GFP. #, P<0.05 between LLC-MUT and LLC-GFP.

WSX1 has an intracellular Box1 motif necessary to interact with JAK1 although JAK2 has been shown to induce downstream signaling [14], [15], [16]. Mutating conserved prolines in the Box1 motif or adding JAK inhibitors attenuates the downstream signaling of WSX1, such as phosphorylation of STAT1/STAT3/STAT5 [17]. Our previous data suggested that WSX1 inhibited tumor growth independently of IL27 signaling [12]. To determine the dependency of WSX1 promotion of tumor growth on IL27 signaling, we first assessed tumor growth differences between LLC cells expressing full-length WSX1 (LLC-WSX1) and WSX1-Mutant (LLC-MUT, lacking intracellular domain including Box1 domain). If LLC-MUT-expressing tumors grew slower than full-length WSX1 in wildtype mice, then it indicates that IL27 promotes tumor growth; otherwise, IL27 does not explain pro-tumorigenic abilities of WSX1. To confirm that IL27 induces signaling only in LLC-WSX1 cells but cannot signal in control LLC cells (GFP expressing cells) and LLC-MUT cells, STAT1 phosphorylation and STAT1 expression (trademarks of the IL27 signaling pathway) were analyzed [18]. As expected, IL27 is able to signal in full-length WSX1 expressing cells, while in WSX1-MUT cells (lacking intracellular domain of WSX1 and Box 1 domain), IL27 has lost the ability to induce phosphorylation and expression of STAT1 (Figure 2a). To confirm that the assay was successful, positive controls of WSX1-MUT ran side by side and in the same gel with full-length WSX1 are included in Figure S1.

*In-vivo*, LLC-MUT-expressing tumors grew faster than either WSX1 or GFP cohorts, excluding the role of IL27 as a tumor promoter (Figure 2b), suggesting that IL27 signaling in tumors may inhibit tumor growth rather than promote tumor growth. This result is in accordance with the result from others, in which IL27 inhibits tumor growth through tumor inhibition of COX2 and PGE2 [19]. But, if IL27 signals in tumor cells and inhibits tumor growth, then why do tumor cells that overexpress WSX1 grow faster than GFP control? One possibility is that tumors overexpressing WSX1 compete with immune cells for IL27 and reduce the bioavailability of IL27 to signal into immune cells since IL27 generates anti-tumor activities via signaling through host immune cells [20], [21], [22], [23]. To exclude this possibility, we compared tumor growth rates between GFP and WSX1-positive LLC tumors in TCCR^−/−^ mice. An increase in tumor growth rates in WSX1-positive tumors compared to the cognate controls in TCCR^−/−^ mice (IL27 cannot signal in this host) suggests that WSX1 expression in tumors does not act as a decoy receptor for IL27 but rather promotes tumor growth via an alternate mechanism. Indeed, similar to wildtype mice, WSX1-expression in tumors promotes tumor growth in TCCR^−/−^ mice (Figure 2c). Moreover, IL27 signaling in WSX1 positive tumor cells (LLC-WSX1) reduces rather than promotes tumor growth (Figure 2d), consistent with other publications [13]. In summary, WSX1's ability to promote tumor growth is not mediated via IL27, but via an alternate mechanism.

### WSX1-mediated tumor growth promotion is not dependent on NKG2D pathway

**Figure 3:**
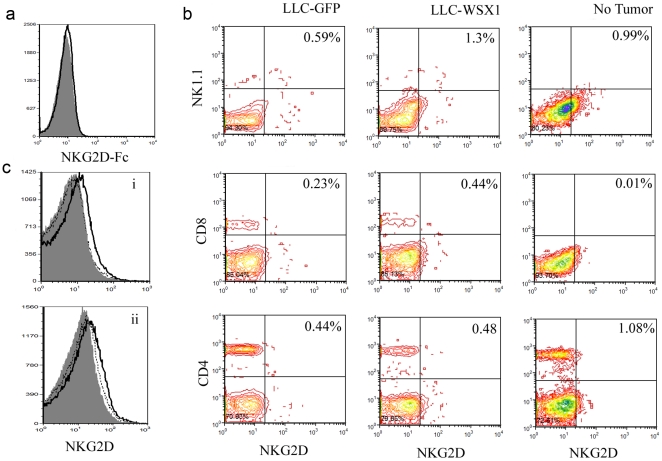
NKG2D-independent and WSX1-dependent tumor growth. (**a**) Comparison of NKG2D ligand expression in LLC cells transduced with either GFP (shaded gray) or WSX1 (not shaded) stained with NKG2D-Fc followed by anti-human IgG-PE. (**b**) Comparison of NKG2D expression in NK^+^, CD8^+^, and CD4^+^ cells in the splenocytes of mice bearing LLC-GFP or LLC-WSX1 tumors, or mice without tumors. Splenocytes from tumor bearing mice were pooled (5 mice/group), processed, and stained with the following antibodies: NK1.1-FITC, CD8-FITC, CD4-APC, and NKG2D-PE. (**c**) Comparison of membrane-only or total cellular expression of NKG2D in the splenocytes of mice bearing LLC-GFP (dotted line) or LLC-WSX1 tumors (solid line) or mice without tumors (shaded in gray). Processed splenocytes were either permeabilized (bottom panel, ii) or left untreated (top panel, i) and stained with NKG2D-PE antibody.

The NKG2D receptor plays an important role in the anti-tumor immune response. Our previous study demonstrates that induction of NKG2D ligands in the WSX1-engineered TC1 and AT84 tumor cells are associated with tumor growth inhibition [12]. However, disruption of the NKG2D pathway leads to the immune escape of tumor cells. It is possible that WSX1 expression in LLC failed to induce NKG2D ligands. As expected, WSX1 does not induce NKG2D ligand upregulation in LLC tumor cells where WSX1 promotes tumor growth (Figure 3a). The lack of NKG2D ligands in WSX1-engineered LLC cells could be due to lack of the induction or could be due to a known mechanism, such as NKG2D ligand shedding by Erp5 [24], [25]. If the shedding mechanism plays a role, then we should observe a suppressed NKG2D expression in immune cells in LLC-WSX1 tumors as compared to the control LLC-GFP tumors, because the shedding of soluble NKG2D ligands downregulates the NKG2D receptor in immune cells [26]. Therefore, we investigated whether WSX1-positive tumors affected NKG2D expression in NK^+^ or CD8^+^ cells *in-vivo*. Our results showed that there is no significant reduction of NKG2D receptors in either NK^+^, or CD8^+^ cells by WSX1 expression in tumor cells (Figure 3b). Moreover, the percentage of immunosuppressive NKG2D^+^CD4^+^ T cells is similar between GFP- and WSX1-positive tumors. Studies comparing the total cellular (cells were permeabilized) or membrane expression of NKG2D in the splenocytes of mice bearing LLC-GFP or LLC-WSX1 revealed that extracellular and total cellular NKG2D expression was increased rather than decreased by WSX1 (Figure 3c) suggesting that internalization of NKG2D is not the mechanism that explains the pro-tumor properties of WSX1 in LLC model. In summary, WSX1-expression in a lung tumor model promotes tumor growth and is associated with a lack of upregulation of NKG2D ligands. The lack of NKG2D ligand upregulation explains why WSX1 does not have the anti-tumor effects but does not explain why WSX1 promotes tumor growth in this tumor model.

### WSX1 triggers immunosuppression in the tumor microenvironment

**Figure 4:**
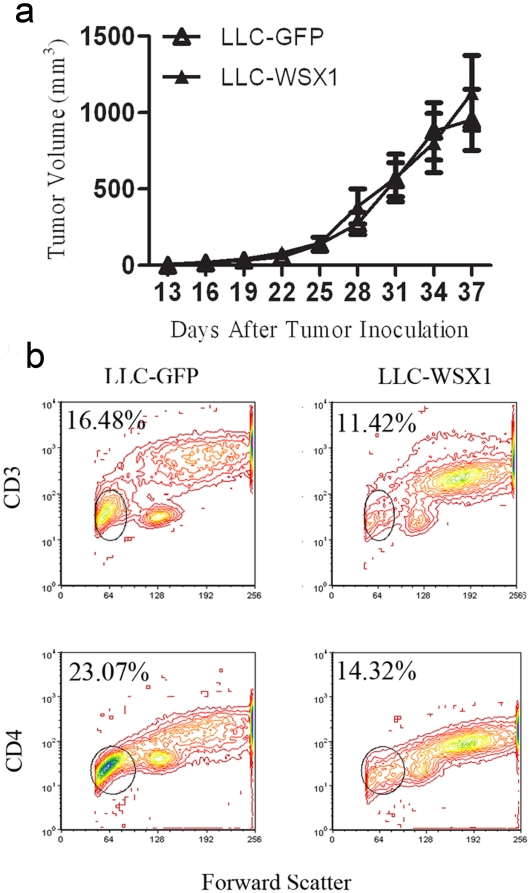
WSX1-mediated immunosuppression in the tumor microenvironment. (**a**) Comparison of tumor growth between LLC-GFP and LLC-WSX1 in nude mice (N = 5–6). (**b**) Comparison of the number of T cells in the tumor microenvironment between LLC-GFP and LLC-WSX1 tumor-bearing mice. Tumors from 5 mice were pooled, processed, and then stained with CD3-APC or CD4-PE. Circled area represents T cell population. Points, mean; bars, SE. *, P<0.05.

WSX1 inhibits tumorigenicity and cell proliferation *in-vitro*, while it promotes LLC tumor growth *in-vivo* (Figure 1). The ability of WSX1 to promote LLC tumor growth was associated with a lack of NKG2D ligand induction (Figure 3a) while WSX1's ability to attenuate TC1 tumor growth was associated with induction of NKG2D ligands. The lack of NKG2D ligand induction in LLC-WSX1 explains why LLC-WSX1 tumor growth was not inhibited, but does not explain why these tumors grow faster than GFP control. Such a result prompted us to hypothesize that WSX1 expression in the absence of NKG2D ligand upregulation promotes immunosuppression and tumor growth while the presence of NKG2D signaling overshadows the immunosuppression pathway, resulting in attenuated tumor growth. In other words, such tumor growth differences between LLC-GFP and LLC-WSX1 may be diminished in immunocompromised mice, in which WSX1 is unable to suppress immune cell surveillance and tumor growth is the same with or without WSX1 expression. To test this hypothesis, we compared tumor growth rates between a control and WSX1-expressing tumors in immunocompromised nude mice. As seen in Figure 4a, T cell absence completely abolished the ability of WSX1 to promote tumor growth. Similar results were obtained in SCID mice too (data not shown). To further support this finding, we investigated the phenotype of immune cell infiltration in the tumor microenvironment of immunocompetent mice. The total number of CD3^+^ and CD4^+^ T cells present in the tumor microenvironment was greatly reduced in WSX1-positive tumors (Figure 4b). This observation is not attributed to lack of T cell infiltration in the tumor microenvironment, as adoptive transfer of CFSE-labeled CD3^+^ T cells had similar tumor-infiltrating ability 16 hours post-transfer (data not shown).

### WSX1 induces immunosuppression in a cell contact-dependent manner

**Figure 5 pone-0019072-g005:**
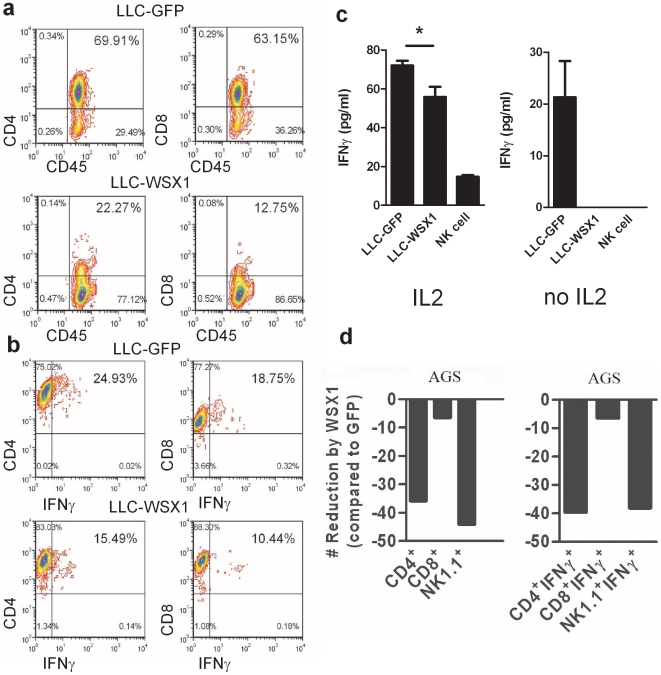
Induction of immunosuppression by WSX1 expression in LLC tumor cells. Comparison of the effect of LLC-GFP vs. LLC-WSX1 (**a**) tumor cells on the number of immune cells *in-vitro*. Splenocytes were co-incubated with the tumor cells mentioned above in the presence of anti-CD3 and anti-CD28 antibodies for 72 hours and stained with the following antibodies: CD45-FITC, CD8-PeCy7, and CD4-PeCy7 (**b**) IFNγ expression in CD4^+^ and CD8^+^, cells was compared in splenocytes co-cultured with either LLC-GFP vs. LLC-WSX1. Purified NK cells in the presence or absence of IL2 (**c**, left and right, respectively) were cultured in the presence or absence (control) of the indicated tumor target cell lines, and IFN-γ release was determined by specific ELISA after 24 hours. Detection of the effects of AGS-GFP vs. AGS-fWSX1 on immune cells. Splenocytes and AGS tumor cells were mixed and analyzed as in (**a**, **b**).

To further characterize how WSX1 reduces the number of immune cells, tumor cells expressing WSX1 or GFP were directly co-incubated with splenocytes activated with CD3/CD28 antibodies. Similar to the *in-vivo* results, the frequency of CD3^+^, CD4^+^, CD8^+^, and NK^+^ cells was reduced by 40–60% in LLC-WSX1 tumors compared to LLC-GFP in culture systems (Figure 5a).

A hallmark of T and NK cell activation is the expression of IFNγ. To determine if WSX1 expression in tumor cells affects their ability to secrete IFNγ, tumors and splenocytes were co-incubated and the percentage of CD4^+^ and CD8^+^ cells expressing IFNγ was determined. The percentage of IFNγ^+^CD4^+^ and IFNγ^+^CD8^+^ cells was also largely decreased in the presence of LLC-WSX1 (Figure 5b) compared to the presence of LLC-GFP. Furthermore, upon co-culturing with LLC-WSX1, NK cells produced lower levels of IFN-γ as compared to the co-culture with LLC-GFP (Figure 5c, left). Similar results were also seen in co-cultures of tumor and NK cells in the absence of IL2 (Figure 5c, right). Thus, the presence of WSX1 in tumor cells directly reduced the production of IFN-γ by NK cells. Likewise, similar results were seen in AGS tumor model (Figure 5d).

**Figure 6 pone-0019072-g006:**
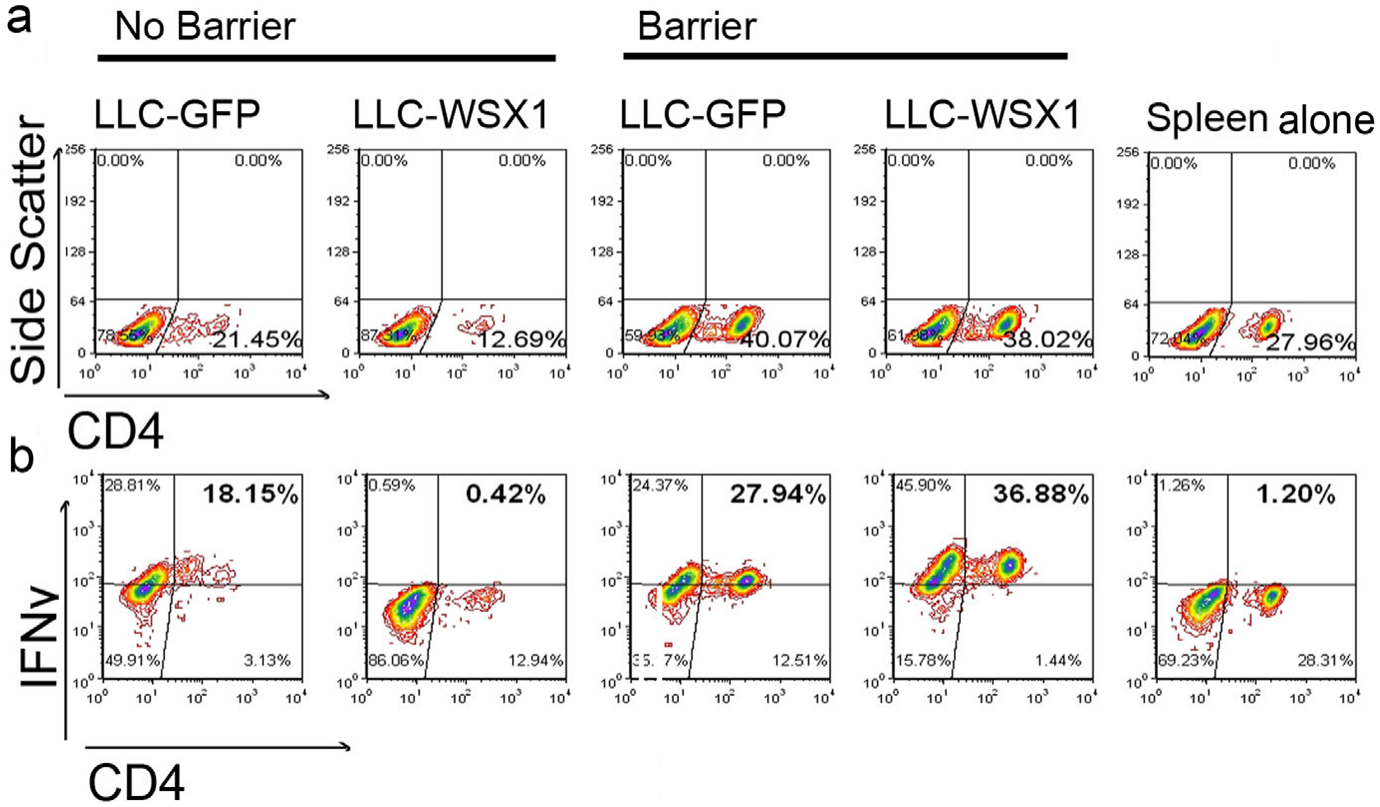
Cell contact-dependent and WSX1-mediated immunosuppression. Detection of the effect of LLC-GFP or LLC-WSX1 tumor cells on CD4^+^ T cells number (**a**) and IFNγ expression (**b**). Splenocytes and tumor cells were either mixed and seeded either in the bottom of the transwell (NO BARRIER) or separated and seeded in the top and bottom of the transwell, respectively (BARRIER). The splenocytes without tumor cells were used as control (SPLEEN). Cells were analyzed similarly as in Figure 5a.

Although WSX1 affects T and NK cell number and cytokine production, we wanted to assess whether WSX1 initiates immunosuppression in a contact-dependent manner or via release of secretable factors. To address this question, LLC-tumor cells where co-incubated with splenocytes directly or separated via a transwell barrier, and T cell number and cytokine production were determined. The use of the transwell barrier reversed the ability of WSX1 to reduce T cell number and IFNγ production in T cells (Figure 6a, 6b), suggesting that WSX1-positive tumors initiate immunosuppression via a direct tumor/immune cell contact-dependent manner.

### WSX1-positive tumors trigger T cell death

**Figure 7:**
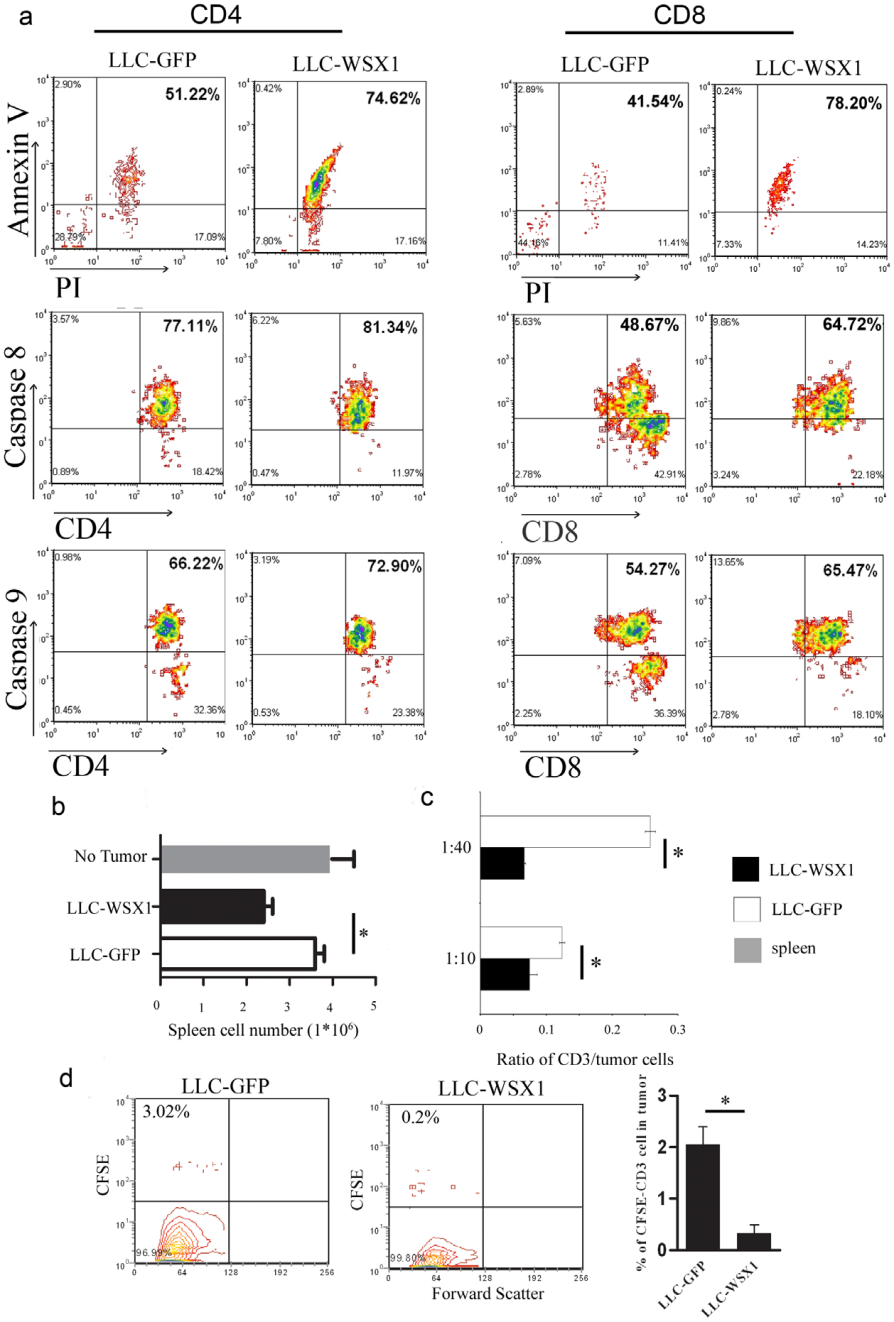
Effect of WSX1 on T cell numbers. (**a**) Comparison of apoptosis markers in CD4^+^ and CD8^+^ T cells when co-incubated with LLC-GFP or LLC-WSX1 tumor cells. Splenocytes and tumor cells are co-incubated as in Figure 5A, and then cells gated for CD4^+^ (left) or CD8^+^ (right) markers were analyzed for Annexin V and propidium iodine (PI) expression, Caspase 8, and Caspase 9. Representative of two independent experiments. (**b**) Comparison the differences in splenocytes cell numbers in the presence of either LLC-GFP or LLC-WSX1 tumor cells. Cells were seeded as in Figure 5A, and viable splenocytes were counted via tryptophan blue exclusion. (**c**) Comparison of purified and CFSE-labeled CD3^+^ T cell numbers in the presence of either LLC-GFP or LLC-WSX1 tumor cells. Data is presented as ratio of gated CFSE-positive lymphocytes (gating based on size in FSC vs. SSC) to tumor cells. (**d**) Detection of CFSE-positive lymphocytes within the tumor microenvironment 6 days post-transfer of tail-vein injection of CFSE- labeled CD3^+^T into LLC-GFP or LLC-WSX1 tumor-bearing mice. N = 4. Representative of two independent experiments. Points, mean; bars, SE. *, P<0.05.

So far, our findings illustrate that WSX1 expression in tumors reduces the number of T cells and IFNγ production by effector immune cells. Next, we aimed to assess how WSX1 reduces T cells population. To address this question, both cell proliferation and cell death were examined in the presence of WSX1-positive or –negative tumor cells. After 72 hours, CD4^+^ and CD8^+^ cells were analyzed for expression of apoptotic markers and cell proliferation via Flow cytometry. WSX1 expression in LLC tumor cells induced elevation of activated caspases 8 and 9 and an increase in percentage of late apoptotic T cells (positive staining for Annexin V and PI) (Figure 7a). Interestingly, a large increase of both activated caspase 8 and caspase 9 (16% and 11.2% increase, respectively) was detected in CD8 T cells when co-incubated with LLC-WSX1 tumor cells as compared to LLC-GFP control cells; meanwhile, relatively small increases of the activated caspase 8 and caspase 9 (4.2% and 6.7% increase, respectively) were found in CD4^+^ T cells. These results suggest that WSX1 expression in tumor cells induces both external and internal cell death signals in T cells, causing cell death. No cell proliferation difference was detected (data not shown).

The observation that WSX1-positive tumors induce T cell death was further confirmed via trypan blue exclusion assay, in which splenocytes were co-cultured with LLC-GFP or LLC-WSX1 and the viable splenocytes were reduced in the presence of WSX1-positive tumor cells as compared to the cognate control cells (Figure 7b).

The results mentioned above do not distinguish whether WSX1-expressing tumors affect T cell number directly or indirectly (via affecting another cell type such as APCs). Thus, purified CFSE-labeled CD3^+^ cells were directly co-incubated with tumor cells expressing either GFP or WSX1 at different ratios. After 48 hours, the ratio of CD3^+^ cells to tumor cells was significantly reduced by WSX1-positive tumor cells (Figure 7c). Such reduction was also shown in an *in-vivo* model, as systemic transfer of CFSE-labeled CD3^+^ T cells was significantly reduced in the tumor microenvironment of LLC-WSX1 when compared to control GPF tumor-bearing mice 6 days post-transfer (Figure 7d).

## Discussion

Mechanisms of tumor escape from immune-facilitated destruction include many evasive strategies. Tumor-associated T cell tolerance and T cell death is induced by several immune suppressive molecules secreted or expressed by tumor associated macrophages, myeloid-derived suppressor cells, tolerogenic DC cells, Treg cells, or tumor cells in the tumor microenvironment [27], [28], [29]. This study reveals that WSX1 expression in tumors negatively regulates immune response in the tumor microenvironment via inducing apoptosis of T cells, and the receptor might be a new target in immune therapy to reverse tumor tolerance in the tumor microenvironment.

The underlying mechanisms by which known immune suppressive molecules regulate immune suppression have been previously explored. In multiple tumors, upregulation of PD-L1 and subsequent binding to its receptor expressed in activated T cells induces apoptosis, anergy, and exhaustion of effector or memory T cells [4], [5], [6]. Upregulation of IDO, a tryptophan-degrading enzyme, induces tryptophan depletion in the local surroundings, which severely downregulates different facets of immune responses, such as effector T cell populations, DC cells, and NK cells [7], [8]. In addition, another well-documented phenomenon associated with T cell death is upregulation of FASL in solid tumor cells, which activates the receptor FAS and intracellular signaling cascade in T cells, resulting in T cell death [30], [31].

In this study we reveal that similar to the immunosuppressive molecules such as PD-L1 and FASL, WSX1 is a novel modulator of the cross-talk between tumor and immune cells, which results in inhibition of IFNγ production from NK and T cells and induction of apoptosis of T effector cells (Figs. 4–7). Multiple lines of *in-vivo* and *in-vitro* evidence shown in this study support this conclusion. First, *in-vitro* co-incubation of WSX1-positive tumor cells reduces the number of T cells and induces a significant increase in caspase 8 and caspase 9 activation as well as an increase in late apoptosis markers mainly in CD8, and to a lesser extent, in CD4 T cells. Second, the number of intravenously injected CFSE-positive T cells is significantly reduced 6 days post-transfer in the tumor microenvironment in LLC-WSX1 tumor cells when compared to control cohorts. The decreased presence of CFSE-positive T cells in the tumor microenvironment is not due to T cell infiltration in tumors, as the number of CFSE-labeled T cells after 16 hours post-injection were similar between GFP and WSX1- positive tumors (data not shown). Third, and most importantly, WSX1 cannot promote tumor growth in immune-compromised nude mice, which once again confirms the ability of WSX1 to induce immune tolerance (Figure 4a). Moreover, aberrant T cell activation plays a key role in multiple autoimmune and chronic inflammatory diseases, showing the therapeutic potential of WSX1 in other disease models. Indeed, transgenic mice expressing high levels of WSX1 in MLR/*lpr* background rendered the autoimmune-prone mice protected from the development of autoimmune disease [32]. Such evidence, combined with our study, suggests that WSX1 plays a key role in downregulating the immune response in multiple disease models.

A hallmark of an effective immune response is the ability of T and NK cells to effectively secrete IFNγ. Secretion of IFNγ plays a key role in macrophage activation, inflammation, T helper 1 (Th1) cell responses, tumor surveillance, immunoediting and host defense against intracellular pathogens [33]. WSX1-positive tumor cells reduce not only the number of T cells but also the percentage of T and NK cells secreting IFNγ, similar to the effect of CTLA and PD-1 ligands [4], [34], [35]. Such inhibition is also observed in both tested tumor models (Fig. 4, 5). These observations of tumor and T cell communication are in line with discoveries in other disease models. For example, others found that WSX1 absence will induce hyperproliferation of T cells after infection with *Trypanosoma cruzi* [36], suggesting that the presence of WSX1 is needed to suppresses T cell hyperproliferation after the infection.

IL27 has distinct features in the immune response and various disease models, and these features vary from pro- to anti-inflammatory. Some of the discordant functions seen by IL27 are due to the model used to study IL27 by either using IL27 knockout models used (WSX1^−/−^, EBI3^−/−^) or overexpression of IL27. For example, in ConA-mediated hepatitis model, the lack of WSX1 worsens the hepatitis score, while a lack of EBI3 ameliorates the hepatitis score [37], [38]. Similar differences were also observed in an arthritis model: in a CIA model administration of IL27 systemically improves the clinical score, while in a proteoglycan-induced arthritis model the lack of WSX1 also improves the clinical scores [39], [40]. The dissociation that is seen in the autoimmune disease models is also observed in cancer-related biology. Previous studies have shown that IL-27 expressing tumors decrease tumor volume by enhancing NK and CTL activity and inhibiting COX-2 expression [19], [20], [21]. In contrast, other studies in conjunction to this study have shown that WSX1-expressing tumors can either promote or inhibit tumor growth independently of IL27 [12], [17]. Similarly, IL27 plays a role not only in tumor growth but also in metastasis. In lung carcinoma, IL27 down-regulates vimentin expression and reduces cellular migration and invasion. IL27 also inhibits metastasis in the B16F10 and neuroblastoma TBJ tumor models [19], [41], [42]. Meanwhile, EBI3 knockout mice are less prone to experimental metastasis of B16F10 [43]. To reconcile the opposing properties of IL27 and WSX1 phenotypes in immune-related diseases or cancer, it is likely that, in addition to WSX1 serving as a receptor for IL27, WSX1 has properties independently of IL27.

Indeed, the findings presented here suggest that WSX1 has a function independent of IL27. Direct co-incubation of WSX1-positive tumor cells and purified CD3^+^ T or NK cells inhibits T cell numbers or NK cell's ability to produce IFNγ whereas neither T, neither NK, nor tumor cells express IL27. More importantly, tumor cells expressing mutated WSX1 (Figure 2) or subcutaneously inoculated in TCCR^−/−^ (Figure 2) grow faster than control cohorts, supporting the notion that inhibition of IL27 signaling in either the host or the tumor does not affect the pro-tumorigenic abilities and induction of tumor tolerance by WSX1.

The fact that WSX1 suppresses the immune system perfectly explains why this tumor growth was promoted when WSX1 expression was elevated by genetically engineered LLC cells. However, it does not explain why the growth of TC1 and AT84 tumors reported previously [12] were inhibited rather than promoted. The most likely explanation is due to differences in tumor cell type and the differences in the intrinsic immune evading mechanisms of each cell type. Another likely explanation is that WSX1 induces both activating and inhibitory signals in cancer cells and the sum of these signals determines the outcome of the immune response and tumor growth. The opposite effect (anti-tumor effect) is determined by the presence of a strong group of immune surveillance signals in the TC1 and AT84 tumor cells following the genetically engineering with WSX1. It is possible that many positive immune stimulation signals were induced in TC1 and AT84 and one group of these immune surveillance stimulation signals are NKG2D ligands, which were detected. Indeed, NKG2D ligands were detected in TC1 and AT84 but were not detected in LLC and Ags tumor cells following the WSX1 genetic engineering. This difference offers a clue but not the final mechanism. In other words, NKG2D ligands alone may not represent the entire immune stimulation signals but just acts as a marker to distinguish which role WSX1 will play. Unfortunately, this paradox needs additional comprehensive studies to be resolved.

In recent years, it has become increasingly evident that lack of successful anti-cancer therapies is mainly due to the immunosuppressive environment created by cancer cells. This study not only demonstrates a novel molecular mechanism for the suppression of immune responses in cancer via inducing T cell death and inhibition of pro-inflammatory cytokine production but also presents a molecular link of the crosstalk between cancer and T cells via WSX1.

## Materials and Methods

### Cell culture and reagents

Mouse cancer cell lines AGS and LLC were kindly provided by Dr. William E. Carson (The Ohio State University, Columbus, Ohio) and Dr. Augusto C. Ochoa (Louisiana State University, School of Medicine, New Orleans, LA), respectively. Other reagents were purchased commercially, which include recombinant mouse IL27, NKG2D/Fc, recombinant human IL2, and anti-CD3 (R&D Systems, Minneapolis, MN, USA); anti- pSTAT1-701, anti-STAT1, anti-hamster IgG-FITC, and anti-hamster IgG-APC (Santa Cruz, Santa Cruz, CA, USA); anti-NKG2D-Pe, anti-NK1.1-FITC, anti-CD4-APC, anti-CD4-FITC, and anti-CD28 (Biolegend, San Diego, CA, USA); anti-CD8-FITC (BD Biosciences, Chicago, IL, USA), and anti-IFNγ-APC, CD45-FITC, CD8-PeCy7, and CD4-PeCy7 (eBiosciences, San Diego, CA, USA). Mouse anti-WSX1 was provided by Dr. Fred de Sauvage (Genentech, San Francisco, CA, USA).

### Flow Cytometry

Cells were stained with the indicated antibodies for 30 min at 4°C as indicated in each figure. The expression of the indicated genes was analyzed on FACS Calibur (BD Biosciences, San Jose, CA, USA) and FCS Express 3 (De Novo Software, Los Angeles, CA, USA). For intracellular staining, BD Biosciences intracellular kit was used according to manufacturer's instructions. Annexin V and activated caspase 8 and 9 (Biovision, Mountain View, CA, USA) staining were performed according to manufacturer's instructions.

### Cell proliferation, soft agar growth assay, and western blot

These assays were performed per protocol as previously described [12].

### Statistical analysis

For *in-vivo* experiments, Univariate Repeated Measures ANOVA was used to analyze the difference among treatments using SAS version 9.1.3. When appropriate, Tukey's HSD test was performed for interaction affects. For *in-vitro* results, student's T test analysis was conducted.

### Cell harvesting, purification, and CFSE staining

Spleens were mashed through a 70 µm cell strainer (Fisher, Pittsburgh, PA, USA) to obtain single cell suspensions. CD3^+^ T and NK cells were purified using negative selection according to manufactures instructions (Stem Cell Separation, Vancuver, BC, Canada). Splenocytes or CD3^+^ T cells were labeled with 20 µM CFSE for 15 minutes at 37°C (Invitrogen, Carlsbad, CA). Tumor-infiltrating lymphocytes were obtained by removing tumors, chopping them into pieces, and resuspending the mixture in sterile PBS (without Ca^2+^ and Mg^2+^) in the presence of a digestion enzyme mixture of collagenase IV, hyaluronidase V, and DNase II (Fisher).

### Confocal microscopy

TC1 and LLC cells expressing fWSX1 were seeded onto glass slides and fixed. The slides were visualized and photographed using Leica TCS SP2 confocal microscope with the help of Dr. Xiaochu Wu (Louisiana State University).

### Adoptive T cell transfer

5*10^6^ CFSE-labeled CD3^+^ T cells were suspended in 100 µL of PBS and injected i.v. into mice bearing LLC-GFP or LLC-WSX1 tumors at 300 mm^3^. The mice were sacrificed 16 hours and 6 days post adoptive transfer, tumors were harvested and processed via enzyme digestion, and CFSE-positive cells in the tumor microenvironment were documented via flow cytometry.

### Establishing stable WSX1 and IL27-signaling defective WSX1 (WSX1-MUT) expressing cell lines

The murine *WSX1* full length gene (gene bank # BC032878) was purchased from Open Biosystems (Huntsville, AL, USA) and subcloned into *pBMN-GFP* (Phoenix™ Retrovirus Expression System) for generating bicistronic gene expression system, the *WSX1-IRES-GFP* retroviral construct. The murine *WSX1* full length gene was first subcloned into *pDsRed-Express* vector (Clontech Laboratories, Mountain View, CA, USA) to make the *WSX1-DsRed* fusion gene (referred to as *WSX1* throughout the manuscript). The fusion gene was subsequently cloned into *pBMN-GFP*, resulting in the *WSX1-DSRed-IRES-GFP* retroviral construct. The *Lac Z* gene in *pBMN-Z* plasmid (Phoenix™ Retrovirus Expression System), was replaced with the *WSX1-DsRed* fusion gene (*rWSX1*) from above for generating the *WSX1-DSRed* retroviral construct. *WSX1* without cytoplasm domain was PCR amplified from *WSX1* gene and subcloned to *pDsRed-Express* to make the *WSX1-MUT-DsRed* fusion gene (*WSX1-MUT*). The fusion gene (*WSX1-MUT-DsRed*) lacking the cytoplasm domain of *WSX1* was subsequently cloned to *pBMN-GFP* for generating the *WSX1-MUT-DSRed-IRES-GFP* retroviral construct. The retrovirus was generated by transfecting *WSX1-IRES-GFP*, *WSX1-MUT-DSRed-IRES-GFP*, or control *GFP* and *DsRed* constructs into Phoenix Eco packaging cells. LLC and AGS cells were transduced with retrovirus containing the gene of interest and the positive fluorescent cells were sorted using a BD FACS Aria III cell sorter.

### *In-vitro* co-incubation assays

Both T cells and splenocytes were used for co-incubation with tumor cells. For T cells, 1*10^6^ CFSE-labeled CD3^+^ T cell were co-incubated with 1*10^5^ or 2.5*10^4^ tumor cells in a 12-well plate for 48 hours in total of 2 mL media, and cells were analyzed via FCS Express Software. For splenocyte co-incubation study, 2*10^6^ splenocytes and 2*10^5^ tumor cells were mixed and seeded. Splenocytes or CD3^+^ T cells were activated with anti-CD3 and anti-CD28 antibodies at concentrations of 2.0 and 0.5 µg/ml, respectively. NK cells were isolated from C57BL/6 splenocytes according to the manufacturer's instructions (Stem Sep) and expanded for 3 days with 4000 U/ml human IL-2. A total of 3*10^4^ NK cells was cultured 1*10^4^ tumor target cells in the presence of 1000 U/ml human IL-2 for 24 h. Concentrations of mouse IFN-γ in the supernatants were determined by ELISA (eBiosciences).

### Animal procedures

Six- to eight-week-old C57Bl/6 and C57Bl/6-WSX1^−/−^ (TCCR^−/−^) mice were used for this study with the approval from IACUC at Louisiana State University, LA (08-098) and The University of Texas M.D. Anderson Cancer Center, TX (03-10-01031). The tumor models were generated by subcutaneously inoculating LLC and AGS tumor cells (2×10^5^ in a 30-µL volume per mouse) into mice. Tumor measurement and calculation were the same as described previously (Puisieux *et al.*, 1998). TCCR KO mice were provided by Dr. Fred de Sauvage (Genentech), while other mice were purchased from commercial sources.

## Supporting Information

Figure S1 Full length blots of cropped gels shown in Figure 2a ran side by side with a positive control.
(TIF)
