# Supplementary figures and images for: WSX1 Expression in Tumors Induces Immune Tolerance via Suppression of Effector Immune Cells
Source: PLoS One. 2011 Apr 29;6(4):e19072. doi: 10.1371/journal.pone.0019072 (PMC3084744; doi:10.1371/journal.pone.0019072)

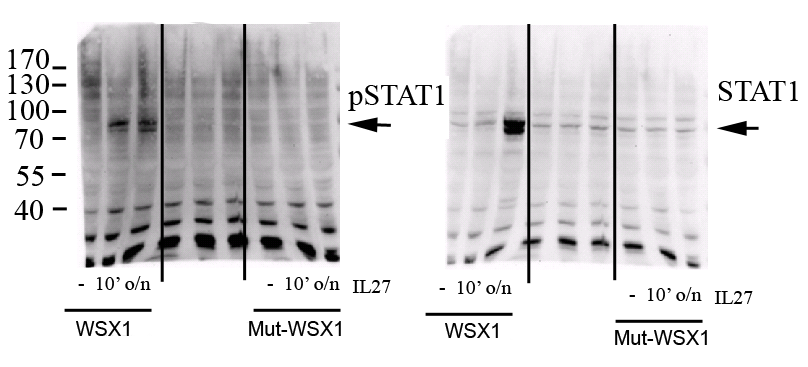

Supplement: Figure S1 — Full length blots of cropped gels shown in Figure 2a ran side by side with a positive control. (TIF) [file pone.0019072.s001.tif]
